# Successful Treatment of Mucormycosis Caused by Rhizopus arrhizus With Amphotericin B and Itraconazole

**DOI:** 10.7759/cureus.35814

**Published:** 2023-03-06

**Authors:** Chen Yi Cham, Anuradha P. Radhakrishnan, Chee Yik Chang

**Affiliations:** 1 Internal Medicine, Hospital Selayang, Selayang, MYS; 2 Infectious Diseases, Hospital Selayang, Selayang, MYS

**Keywords:** rhino-orbital mucormycosis, itraconazole, amphotericin b, rhizopus arrhizus, mucormycosis

## Abstract

Mucormycosis is a serious and often fatal fungal infection that is most commonly observed in immunocompromised individuals. The mortality rate of mucormycosis is high if left untreated, and successful treatment requires a combination of antifungal therapy, surgical intervention, and reversal of the underlying immunocompromised state. The choice of antifungal treatment is crucial and depends on several factors, including the safety profile of the drug, its spectrum of activity, and the species of fungus causing the infection. In this report, we describe a case of a patient who presented with mucormycosis and was successfully treated with a combination of antifungal therapy, surgical excision of affected tissue, and reversal of the underlying immunocompromised state. Our report underscores the importance of early recognition and aggressive treatment of mucormycosis to improve outcomes for affected patients.

## Introduction

Mucormycosis is an angio-invasive fungal infection caused by fungi of the class *Zygomycetes* in the order *Mucorales*, with rhino-cerebral mucormycosis being the most common form of the disease. The infection primarily affects immunocompromised individuals and carries a high mortality rate if left untreated. Risk factors for mucormycosis include diabetes mellitus, prolonged steroid use, malignancy, hematological malignancy, and post-transplantation [1]. Diagnosis of mucormycosis remains a challenge due to the requirement of high clinical suspicion and the need for minimally invasive tissue biopsy procedures followed by identification of the fungal material by molecular techniques [2].

Antifungal therapy is the mainstay of treatment, with amphotericin B being the antifungal of choice for decades. However, the side effects of amphotericin B can be detrimental, and newer broad-spectrum antifungal agents such as posaconazole and isavuconazole are emerging as potentially effective treatment options [3]. Furthermore, the high treatment cost of posaconazole may limit its availability, particularly in resource-limited settings. Here, we report a case of mucormycosis in a diabetic patient who was successfully treated with amphotericin B and itraconazole along with extensive debridement of the diseased tissue.

## Case presentation

A 64-year-old female with a medical history of type 2 diabetes mellitus presented to Hospital Selayang, Selayang, Malaysia, in March 2022 with a chief complaint of right eye redness accompanied by right-sided headache and photophobia over the course of two weeks. One week prior to admission, the patient reported right nasal discharge and pain. She denied any history of preceding trauma or falls and reported residing in a flat equipped with air conditioning, with no contact with soil.

**Figure 1:**
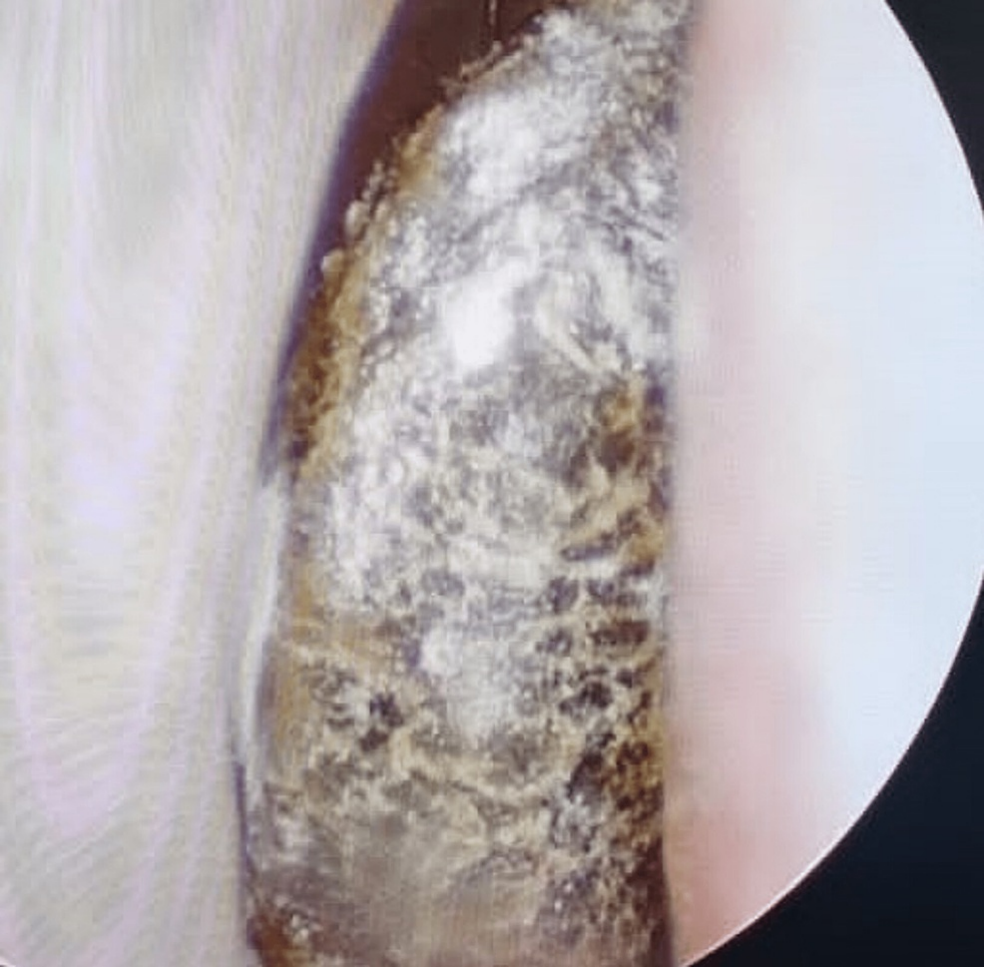
Rigid nasal endoscopy revealed eschar in the middle turbinate

On examination, the patient was afebrile with a blood pressure of 124/63 mmHg and a pulse rate of 99 beats per minute. There was no evidence of respiratory distress, and the patient's oxygen saturation was 99% while breathing ambient air. Notably, no facial asymmetry, conjunctivitis, proptosis, or ophthalmoplegia was observed. Additionally, a neurological examination did not reveal any meningism, neurological deficits, or cranial nerve palsies. A rigid nasal endoscopy demonstrated eschar in the middle turbinate (Figure 1), while the otoscope examination was unremarkable with intact tympanic membranes.

The patient's blood investigation results showed leukocytosis with a total white cell count of 16 x 10^9^/L, anemia with a hemoglobin level of 9.8 g/dL, and mild reactive thrombocytosis with a platelet count of 472 x 10^9^/L. The full blood picture showed ovalostomatocytosis and the presence of target cells, along with neutrophilia, which suggested the presence of an acute infection. The renal and liver function tests were within the normal range.

**Figure 2:**
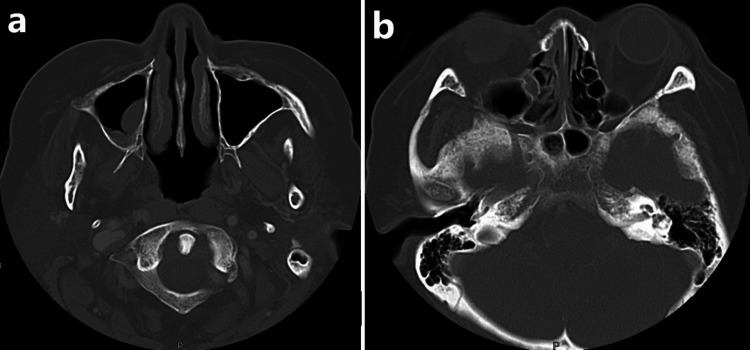
CT of the paranasal sinuses (axial view) revealed mucosal thickening of the right maxillary and sphenoid sinuses with surrounding sphenoid bone thickening

Further imaging through a computed tomography (CT) scan of the paranasal sinuses revealed mucosal thickening in the right maxillary and sphenoid sinuses, along with surrounding sphenoid bone thickening, which was indicative of right maxillary and sphenoid sinusitis (Figure 2). Additionally, a cranial CT scan detected a well-defined hypodensity at the left external capsule, consistent with chronic infarction.

**Figure 3:**
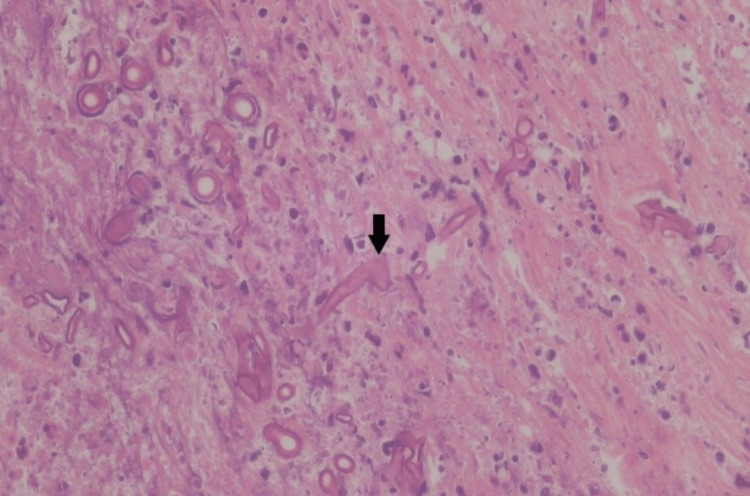
Histopathological examination of the lesion revealed wide-septated fungal hyphae (indicated by the arrow)

On the fourth day of hospitalization, the patient underwent a right medial antrostomy and middle turbinectomy. During the surgery, an eschar was observed in the right middle turbinate, along with a diseased medial wall of the right maxillary sinus. A biopsy of the affected tissue was taken and sent for histopathological examination. The results revealed the presence of wide-septated fungal hyphae with necrosis, as confirmed by positive staining for fungal elements on both periodic acid-Schiff and Grocott methenamine silver staining (Figure 3). Further testing using a fungal polymerase chain reaction (PCR) identified *Rhizopus arrhizus* as the causative organism. Susceptibility testing was subsequently performed, which showed that itraconazole had the lowest minimum inhibitory concentration (MIC) of 0.12 µg/mL, followed by posaconazole and amphotericin B, both with MICs of 0.25 µg/mL.

After the diagnosis of mucormycosis, the patient was started on intravenous amphotericin B deoxycholate for a planned total duration of six weeks. However, due to the development of electrolyte imbalance and acute kidney injury despite hydration protocol, amphotericin B had to be discontinued on day 35. As posaconazole and isavuconazole were not available in the hospital, oral itraconazole was initiated as an alternative therapy. The patient responded well to the treatment, and a repeat nasal endoscopy was performed, which revealed no residual fungal debris.

The patient was followed up at the outpatient clinic and completed a three-month course of oral itraconazole. As of the time of writing, she was still asymptomatic with no signs of disease relapse. The successful management of this case highlights the importance of a high index of suspicion for mucormycosis in high-risk patients, as well as the need for prompt and appropriate antifungal therapy and adequate surgical debridement.

## Discussion

Mucormycosis is a rare but rapidly spreading invasive fulminant fungal infection of the order *Mucorales*, class *Zygomycetes.* The majority of clinical specimens recovered are from the *Rhizopus* genera, and *Rhizopus arrhizus* accounts for 60% of all forms of mucormycosis, making it the most common human pathogen overall [2]. The *Mucorales *are found in decaying organic matter, soil, contaminated foods, and construction work, as well as contaminated air filters and aerosolization remains the primary mode of transmission [4].

Several risk factors are associated with mucormycosis. These include diabetes mellitus, hematological malignancies, and bone marrow or solid organ transplantation [1]. A case report of rhino-orbital mucormycosis in a COVID-19 patient was previously reported in Malaysia [5]. Additionally, the use of deferoxamine chelation therapy can increase the risk of infection, as *Rhizopus* sp. can use it as a siderophore to obtain iron and enhance growth [6]. Lastly, the use of voriconazole for treatment or prophylaxis in stem cell transplant recipients may also play a role in the pathogenesis of mucormycosis [7].

Mucormycosis is an aggressive and angio-invasive infection that spreads rapidly, leading to tissue necrosis. The fungal hyphae invade the arteries and lymphatic systems, causing the formation of "mucor thrombi," which results in further occlusion, infarction, tissue necrosis, and the development of black necrotic eschar. The infection can spread to adjacent sinuses, orbit, and cranium. Based on the site of infection, mucormycosis is classified into six major forms: rhino-cerebral, pulmonary, cutaneous, gastrointestinal, disseminated, and uncommon rare forms such as endocarditis, osteomyelitis, peritonitis, and renal infection [2,5].

Rhino-cerebral mucormycosis presents with symptoms such as headache, rhinorrhea, and epistaxis, and may also cause fever and black eschar formation over the nasal or oral cavity. The infection can invade the maxillary, frontal, and ethmoidal paranasal sinuses, leading to facial numbness and edema. If left untreated, the orbital invasion may cause proptosis, blurred vision, diplopia, orbital pain, and paresthesia. Cerebral invasion is the most severe form of the disease [8]. In our case, invasive mucormycosis was suspected after a rigid nasal endoscopy showed eschar, and a histopathological examination revealed wide septated fungal hyphae with necrosis. The diagnosis was confirmed using polymerase chain reaction detection for* Rhizopus arrhizus*.

The current mainstays of treatment for mucormycosis include amphotericin B, posaconazole, and isavuconazole, in combination with surgical debridement. However, these newer antifungal agents, such as posaconazole and isavuconazole, are expensive and not always readily available [9]. While itraconazole has not been commonly used for the treatment of mucormycosis, its MIC for certain species, such as *Rhizopus arrhizus*, has been reported to be low. However, clinical breakpoints for the *Mucorales* and any antifungal agent have not been established [10]. In our case, the patient responded favorably to itraconazole, which was likely due to the low MIC (0.12 µg/mL) of the organism. Therefore, accurate identification of the species causing the infection is critical to ensuring appropriate management and selecting the most effective antifungal agent. Additionally, timely administration of antifungal therapy is crucial for improving clinical outcomes in invasive fungal infections [9,11].

## Conclusions

In conclusion, this case report highlights the importance of early recognition and prompt management of mucormycosis, which is a rare but potentially fatal fungal infection. The successful outcome of our patient was due to a combination of appropriate antifungal therapy, aggressive surgical debridement, and effective management of the underlying immunocompromised state. It is crucial for healthcare providers to have a high index of suspicion for mucormycosis in immunocompromised patients presenting with symptoms of sinusitis or other invasive fungal infections.
